# Specific and Sensitive Isothermal Electrochemical Biosensor for Plant Pathogen DNA Detection with Colloidal Gold Nanoparticles as Probes

**DOI:** 10.1038/srep38896

**Published:** 2017-01-17

**Authors:** Han Yih Lau, Haoqi Wu, Eugene J. H. Wee, Matt Trau, Yuling Wang, Jose R. Botella

**Affiliations:** 1 Centre for Personalized Nanomedicine, Australian Institute for Bioengineering and Nanotechnology, The University of Queensland, Australia; 2 Plant Genetic Engineering Laboratory, School of Agriculture and Food Sciences, The University of Queensland, Australia; 3 Department of Macromolecular Science, National Key Laboratory of polymer engineering, Fudan University, Shanghai, 200433, China; 4 School of Chemistry and Molecular Biosciences, The University of Queensland, Brisbane, QLD 4072, Australia

## Abstract

Developing quick and sensitive molecular diagnostics for plant pathogen detection is challenging. Herein, a nanoparticle based electrochemical biosensor was developed for rapid and sensitive detection of plant pathogen DNA on disposable screen-printed carbon electrodes. This 60 min assay relied on the rapid isothermal amplification of target pathogen DNA sequences by recombinase polymerase amplification (RPA) followed by gold nanoparticle-based electrochemical assessment with differential pulse voltammetry (DPV). Our method was 10,000 times more sensitive than conventional polymerase chain reaction (PCR)/gel electrophoresis and could readily identify *P. syringae* infected plant samples even before the disease symptoms were visible. On the basis of the speed, sensitivity, simplicity and portability of the approach, we believe the method has potential as a rapid disease management solution for applications in agriculture diagnostics.

Plant diseases are one of the major causes of economic losses for the agricultural industry with losses estimated to exceed USD150 billion annually worldwide^1^. Many disease management strategies have been implemented to control crop losses globally and, in the absence of resistant varieties, early disease detection is paramount to avoid losses and reduce the spread of the disease to neighbouring farms. Therefore, rapid, sensitive and specific diagnostic methods for plant pathogen detection are crucial in facilitating effective disease management practices.

Traditionally, experienced plant pathologists diagnose diseases by observing typical disease symptoms and identify the pathogen by culturing in specialized media^2^. This method is mostly accurate but is time consuming and therefore not suitable for rapid disease management practices. Immunoassays have hence been widely used for plant pathogen detection since the 1980s to address the limitations of symptomatic diagnosis. However, antibody based methods are prone to cross reactivity with closely related pathogen species having similar epitopes for antibody recognition^3^. In addition, antibodies tend to have short shelf life and are prone to variations between production batches^4^. DNA-based molecular diagnostic methods have been proposed to increase the reliability, sensitivity and specificity of plant pathogen detection. The polymerase chain reaction (PCR) is currently the most popular and reliable molecular technique used in plant pathogen diagnostic assays^5^,^6^. However, to avoid the instrumentational limitations of PCR, isothermal approaches such as the recombinase polymerase amplification (RPA)^7^ which uses enzymes instead of heat to achieve exponential amplification have been developed. With PCR-like sensitivity, yet requiring only a low constant working temperature, RPA is potentially useful for point-of-care (POC) applications^8^,^9^,^10^,^11^,^12^. However, there is still a need to develop simpler RPA assays for plant pathogen detection in the field comprising all steps, from DNA isolation to visualization of results.

Electrochemical DNA biosensors have been widely used in disease diagnosis as they potentially offer high sensitivity, rapid analysis and portability at potentially lower cost than traditional technologies^13^. For this reason, the use of screen printed electrodes has gained popularity in POC development platforms. Several DNA biosensors have been reported in previous studies which involve the labelling of PCR products with enzymes^14^, redox active components^15^ or nanoparticles^16^,^17^ to enhance the electrochemical signal. Of the nanoparticle-base strategies, gold nanoparticles (AuNPs) are the most widely used and have gained significant attention recently as an electrochemical label. This is because they provide a large surface area for chemical reactions and have outstanding surface immobilization advantages which can enhance the efficiency of the electrochemistry detection assay^18^,^19^,^20^. Therefore, AuNPs-based DNA biosensors can provide a promising platform for the development of rapid, sensitive, specific and portable diagnostic tools for detecting DNA^21^,^22^,^23^.

Herein, we describe a rapid and highly sensitive diagnostic method coupling RPA with AuNPs as electrochemical probes to detect the presence of plant pathogen DNA by differential pulse voltammetry (DPV) on disposable screen printed carbon electrodes (SPCE). Simultaneously, the performance of our assay was compared with conventional PCR and gel electrophoresis. *Pseudomonas syringae* was used as a model system in this study because it infects wide variety of crops and is an economically important plant pathogen^24^. In the plant kingdom, a singleplex detection method is sufficient for plant disease detection because the plant death is unlikely caused by multiple pathogens infection simultaneously^1^. Therefore, this singleplex diagnostic method might bring to a significant contribution in plant disease detection. Besides, we believe that this assay can have wide applications to detect other DNA targets in agriculture and human diagnostics.

## Materials and Methods

### Reagents and materials

#### Plant and pathogen materials

*Arabidopsis thaliana* ecotype Columbia (Col-0) was obtained from the Arabidopsis Biological Resource Center (ABRC; Ohio State University). *Pseudomonas syringae* DC3000 (Psy) was obtained from the Department of Agriculture, Fisheries and Forestry, Queensland, Australia.

#### Materials and Chemical reagents

KAPA2G Robust HotStart PCR kit was purchased from KAPA Biosystems (Cat#KK5516). Streptavidin coated magnetic beads were obtained from New England Biolabs (Cat#S1420S). Oligonucleotides were synthesized by Integrated DNA Technologies. TCEP (tris(2-carboxyethyl)phosphine), PBS (Phosphate-buffered saline) and Tween 20 (Polysorbate 20) were from Sigma-Aldrich. Screen-printed carbon electrodes (SPCE, DS 110) were purchased from Dropsens.

### Instrumentations

Bio-Rad thermo cycler (MJ Mini Personal Thermal Cycler) and 254 nm transilluminator (Vision-capt version 14.2) were used for PCR amplification and PCR products visualization. DPV responses were measured on a workstation potentiostat (CH Instruments).

## Methods

### DNA sample preparation

The *Pseudomonas syringae* infected Arabidopsis plants were prepared according to literature study^25^,^26^. *P. syringae* pv *tomato* strain DC3000 was cultured on King’s B plate at room temperature for overnight. The culture plate was washed with 10 mM MgCl_2_ to resuspend the bacteria. Six weeks old *Arabidopsis thaliana* seedlings were sprayed with 2 to 5 × 10^8^ cfu/ml of *P. syringae* suspension in 10 mM MgCl_2_ and 0.03% Silwet L-77. The infected leaves with different symptoms severity were collected after 7 days inoculation^27^. The total genomic DNA were extracted from Stage 1 (before visible symptoms), Stage 2 (just symptoms become visible) infection and healthy (H) leaves using solid-phase reversible immobilization (SPRI) beads as described in previous study^27^.

### Preparation of AuNPs-DNA probe

AuNPs were synthesized by the citrate reduction of HAuCl_4_ according to the reported protocol^28^. A volume of 100 mL 1 mM chloroauric acid (HAuCl_4_) was heated to boil and then added with 10 mL of 1% 38.8 mM sodium citrate (Na_3_C_6_H_5_O_7_). The mixture was heated for another 10 minutes and stirred for 30 minutes. The as-prepared AuNPs were characterized with transmission electron microscopy (TEM; JEOL-2100). The size of AuNPs was approximately 16 nm as shown in TEM image (Fig. S1) To prepare the AuNPs-DNA probe, 100 μl of TCEP and DNA probe mixture was prepared by adding 50 μL of 10 mM TCEP into 50 μL of 100 μM DNA probe and then incubated at room temperature for 2 hours. The mixture was then mixed with 1 mL of freshly prepared AuNPs solution and continued shake at the speed of 45 rpm for overnight at room temperature. After that, the mixture was centrifuged at 17500 × g and 4 °C for 25 min, and then the supernatant was removed. The AuNPs were resuspended in 200 μL of 0.1 M PBS.

### Polymerase chain reaction (PCR)

Amplification of DNA targets were performed in a 25 μL reaction containing 1 μL of extracted genomic DNA (1 ng), 1 unit of KAPA Hotstart DNA polymerase, 1X PCR buffer, 0.2 mM each dNTP and 0.25 μM for each universal forward and reverse primers (Table 1). PCR reaction was carried out in a Bio-Rad thermo cycler using the following conditions: denaturation at 95 °C for 5 min followed by 30 cycles of 95 °C for 30 s, 58 °C for 30 s and 72 °C for 30 s and final elongation at 72 °C for 1 min. The products were validated by gel electrophoresis using 1.5% agarose gels containing GelRedTM 1X staining solution in sodium borate buffer. Then the gel was visualized under a 254 nm transilluminator.

### Recombinase polymerase amplification (RPA)

The TwistAmp Basic RPA Kit (TwistDX) was used as recommended by the manufacturer with some modifications. The RPA reaction were performed in the total volume of 12.5 μL at 37 °C for 20 min using 1 μL of the extracted nucleic acid (1 ng) and 480 nM of primer set (Table 1). The current primer set used in this study performed with no observable differences between 37–42 °C. Beyond this range, loss of amplicon yield was observed, which was consistent with the literature^7^ and with the manufacturers specifications. Finally 2.5 μL of the RPA reaction was verified by gel electrophoresis. 10 μL of RPA product was used in the electrochemistry detection.

### Assay design and fabrication

**Figure 1:**
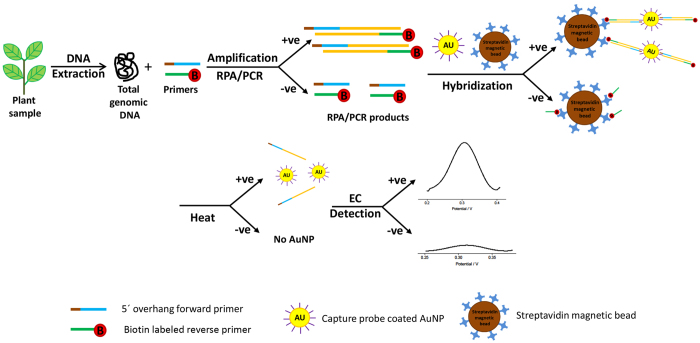
Schematic illustration of the electrochemical bioassay for plant pathogen DNA detection.

Electrochemical bioassay for plant pathogens detection involved first incubation of the amplified target, AuNPs-DNA probes and the streptavidin beads (as shown in Fig. 1). Briefly, 5 μL of AuNPs/DNA probe was mixed with 10 μL of PCR/RPA product and topped by distilled water to be 20 μL in final volume. The mixture was then incubated at 37 °C for 20 min. Next, 5 μL of streptavidin coated magnetic beads were added into the solution and was further incubated at room temperature for 10 min. Beads were then captured by a magnetic plate, and the supernatant was removed. The beads were washed 3x with 50 μl of PBST buffer (1 mM PBS, 0.01% Tween20), and resuspended in 10 μL of 10 mM PBS buffer, followed by 95 °C heating for 5 min in thermal block or hot water in thermal flask. The solution was then transferred to the surface of working electrode on SPCE and air dried for electrochemical read-out.

### Electrochemistry detection

The AuNPs dried on the working electrode was electrochemically activated by adding 100 μL of 0.1 M HCl and pre-oxidized at a potential of 1.3 V for 30 seconds to oxidize Au^0^ to Au^3+^. Then the reduction of this species was analyzed with differential pulse voltammetry (DPV) response. The electrochemistry response was taken from 0.45 V to ending potential of 0.20 V with the incremental potential of 0.004 V, amplitude potential of 0.05 V, and pulse period of 0.2 s.

## Results and Discussion

### Assay design

A flow diagram showing the main steps in our assay for plant pathogen DNA detection is illustrated in Fig. 1. Total genomic DNA was extracted from plant samples using the solid phase reversible immobilization (SPRI) method^29^, followed by mixing with the primers for amplification. Specifically, RPA was used to amplify the target sequence using a biotin labeled reverse primer and a forward primer containing a 10 nt barcode covalently linked to the 5′ end via a carbon spacer. After amplification, the resulting amplicons, containing a 10 nt overhang (barcode sequence) on one end and biotin on the other, were hybridized to AuNPs-DNA tags which carried probes complementary to the barcode sequence. Streptavidin magnetic beads were then used to enrich for AuNPs/DNA/biotin products. This was followed by a wash to remove excess reagents. Finally, In order to prevent the interference of the electrochemical signal from iron ions (magnetic beads), the magnetic beads/AuNPs/DNA/biotin products were heat treated at 95 °C to denature the dsDNA amplicons and to release any bound AuNPs into solution where the electrochemical reduction of Au (III) to Au (0) was measured with differential pulse voltammetry (DPV). The amount of released AuNPs is proportional to the amount of amplified target DNA which, in turn, denotes a successful RPA/PCR amplification and thus indicating the presence of the pathogen. The use of colloidal nanoparticle in detecting *Mycobacterium tuberculosis* had been reported in our previous study^30^. However, the method was further improved to serve the purpose of field application. This assay has been simplified to a method with no blocking and incubation steps required on electrode. Furthermore, the assay is less expensive by using bare screen printed carbon electrode (SPCE) (USD 2.50 per test) compared to streptavidin coated SPCE (USD 5 per test). SPCE without streptavidin coated is more stable at room temperature which makes the assay scheme more attractive for the on-site application.

### Specificity study

**Figure 2:**
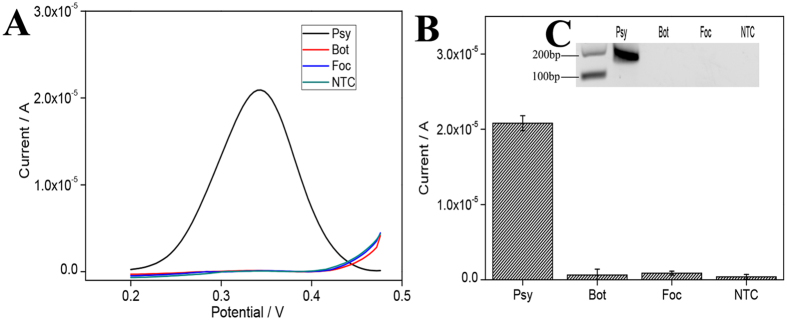
Specificity study for plant pathogen DNA detection. (**A**) DPV curve and (**B**) Current-response to *P. syringae* (Psy), *Botrytis cinerea* (Bot) and *Fusarium oxysporum* f.sp. *conglutinans* (Foc) as well as a no template control (NTC). Error bars represent ±SD, n = 3. (**C**) Electrophoresis gel image of RPA products.

Specificity of a diagnostic method is essential in identifying a particular pathogen from other species to avoid false positive results. To this end, a *P. syringae*-specific assay was challenged with *P. syringae* and two unrelated pathogens: *Botrytis cinerea* and *Fusarium oxysporum* f.sp. *conglutinans* (Fig. 2). As expected, *P. syringae* samples produced a strong DPV signal while no signal was detected from *B. cinerea* and *F. oxysporum*. Electrochemical results were verified using gel electrophoresis where the expected 144 bp band was seen only in the *P. syringae* sample but not the controls. Similarly, the PCR-based assay could also accurately identify the *P. syringae* sample albeit producing a lower DPV signal (Fig. S2).

### Sensitivity studies

**Figure 3:**
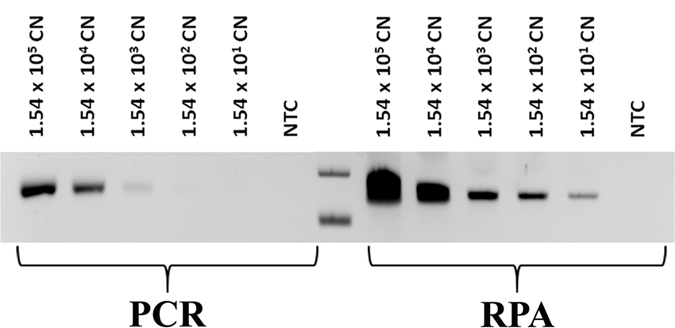
Electrophoresis gel image for the sensitivity comparison between RPA and PCR over a range of gDNA inputs.

To prevent diseases from spreading to neighbouring plants, sensitive diagnostic assays are advantageous for early infection detection even before the symptoms are visible. Hence, to identify a suitable amplification method, we first compared the sensitivity of RPA with PCR (Fig. 3) using same amount of *P. syringae* genomic DNA and primers. It was found that RPA (15 copies) was 100 times more sensitive than PCR (1500 copies) based on gel electrophoresis, and thus a more sensitive yet convenient (by virtue of being an isothermal amplification) alternative to PCR.

**Figure 4:**
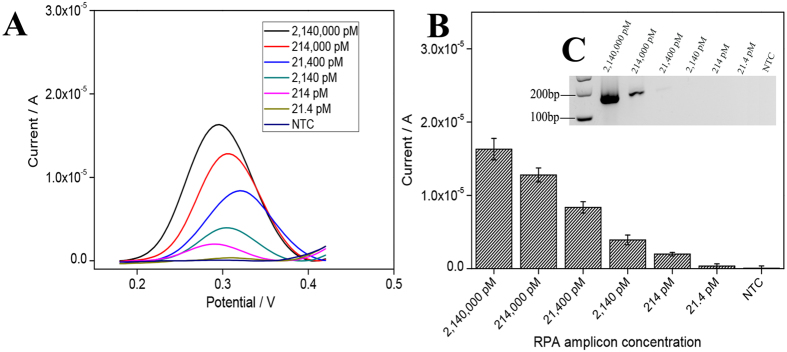
Sensitivity study for plant pathogen DNA detection via RPA/electrochemistry. (**A**) DPV curve and (**B**) Current-response to different amounts of amplification products, the error bars represent ±SD, n = 3. (**C**) Electrophoresis gel image of the RPA products.

Next, the detection limit of the electrochemical assay in detection amplified DNA was determined by titrating RPA products and measuring the corresponding DPV signal (Fig. 4). The data indicated that the electrochemical assay (214 pM) was 100 times more sensitive than gel electrophoresis (21,400 pM). Together, the data thus suggested that the combination of RPA with an electrochemical readout could potentially result in a rapid, sensitive and convenient DNA detection platform. In addition, the relative standard deviation (RSD) of the RPA/electrochemistry assay was 10.7% which indicated good assay reproducibility.

### Electrochemical detection of infected *A. thaliana*

**Figure 5:**
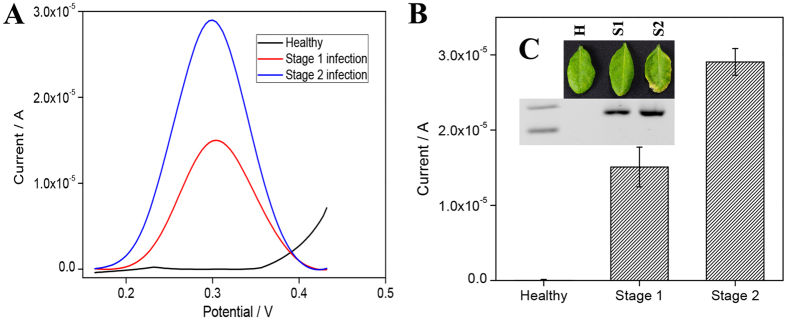
RPA/electrochemistry detection on healthy and *P. syringae* infected *A. thaliana* plant tissue samples (Stage 1 and Stage 2). (**A**) DPV curve and (**B**) Current-response of healthy and infected samples. Error bars represent ±SD, n = 3. (**C**) Images of healthy and diseased *A. thaliana* leaves (Stage 1 and Stage 2) with the electrophoresis gel image of RPA products.

As our initial results with AuNPs-based DNA biosensor were very promising, we wondered if the approach could be applied to complex samples such as diseased plants. To this end, total genomic DNA was extracted from the leaves of a healthy plant and those infected with *P. syringae* at two different degrees of infection (Stage 1 and stage 2) (Fig. 5). Concentration of *P.syringae* in 1 ng of infected plant DNA was determined using qPCR (Fig. S3). The estimated *P.syringae* DNA in Stage 1 and Stage 2 infections were 13 pg and 54 pg, respectively in 1 ng of extracted DNA from infected leaves. With this approach, *P. syringae* was successfully detected disease from as early Stage 1; before symptoms were visible. The DPV signal obtained from Stage 2 sample was two times higher than Stage 1 sample and no signal was detected from the healthy sample. This demonstrated the assay had potential for early detection of pathogens in infected plant samples.

In conclusion, we have successfully developed a highly sensitive method for plant pathogen detection by combining RPA with nanoparticle-electrochemistry. Our results indicated that the electrochemical assay was suitable to detect pathogens with high efficiency, specificity and sensitivity. The biosensor was trialled on *P. syringae* infected *A. thaliana* successfully and could potentially detected infections even before disease symptoms appear. We believe that our method of coupling RPA with an electrochemical readout performed on disposable screen printed electrodes could form the basis of a rapid, sensitive and cost effective POC platform for nucleic acid detecting applications.

## Additional Information

**How to cite this article**: Lau, H. Y. *et al*. Specific and Sensitive Isothermal Electrochemical Biosensor for Plant Pathogen DNA Detection with Colloidal Gold Nanoparticles as Probes. *Sci. Rep.* **7**, 38896; doi: 10.1038/srep38896 (2017).

**Publisher's note:** Springer Nature remains neutral with regard to jurisdictional claims in published maps and institutional affiliations.

## Supplementary Material

Supplementary Information

## Figures and Tables

**Table 1:** Sequences of oligonucleotides involved in this study.

Target/GenBank Accession	*Pseudomonas syringae* pv. tomato str. DC3000 AE016853.1
5′-Forward-3′	TACACAGCAC(C3)TTTGTCCGAAACGACGTACAGCCATTTAACCTT
5′-Reverse-3′	Biotin-TTC TAC GTC GGG GTA TTT ACT AGC TGG AAA AG
Capture probe	GTGCTGTGTATTTTT-SH

## References

[b1] Agrios G. N. Plant pathology. 5th edn, (Elsevier Academic Press, 2005).

[b2] Horsfall J. G. & Cowling E. B. Plant disease: an advanced treatise. (Academic Press, 1977).

[b3] Franken A. A. J. M., Zilverentant J. F., Boonekamp P. M. & Schots A. Specificity of Polyclonal and Monoclonal-Antibodies for the Identification of Xanthomonas-Campestris Pv Campestris. Netherlands Journal of Plant Pathology 98, 81–94, doi: 10.1007/Bf01996321 (1992).

[b4] Murphy K., Travers P., Walport M. & Janeway C. Janeway’s immunobiology. 8th edn, (Garland Science, 2012).

[b5] Ward E., Foster S. J., Fraaije B. A. & McCartney H. A. Plant pathogen diagnostics: immunological and nucleic acid-based approaches. Ann Appl Biol 145, 1–16, doi: 10.1111/j.1744-7348.2004.tb00354.x (2004).

[b6] Vincelli P. & Tisserat N. Nucleic acid-based pathogen detection in applied plant pathology. Plant Dis 92, 660–669, doi: 10.1094/Pdis-92-5-0660 (2008). 30769590

[b7] Piepenburg O., Williams C. H., Stemple D. L. & Armes N. A. DNA detection using recombination proteins. Plos Biol 4, 1115–1121, doi: 10.1371/journal.pbio.0040204 (2006). PMC1475771 16756388

[b8] Ng B. Y. C., Wee E. J. H., West N. P. & Trau M. Rapid DNA detection of Mycobacterium tuberculosis-towards single cell sensitivity in point-of-care diagnosis. Sci Rep-Uk 5, doi: 10.1038/Srep15027 (2015).

[b9] Wee E. J. H., Ngo T. H. & Trau M. Colorimetric detection of both total genomic and loci-specific DNA methylation from limited DNA inputs. Clin Epigenetics 7, doi: 10.1186/s13148-015-0100-6 (2015). PMC4498563 26167236

[b10] Liljander, A. . Field-Applicable Recombinase Polymerase Amplification Assay for Rapid Detection of Mycoplasma capricolum subsp capripneumoniae. J Clin Microbiol 53, 2810–2815, doi: 10.1128/Jcm.00623-15 (2015). 26085615 PMC4540935

[b11] Abd El Wahed A. . Recombinase Polymerase Amplification Assay for Rapid Diagnostics of Dengue Infection. PloS one 10, doi: 10.1371/journal.pone.0129682 (2015). PMC4468249 26075598

[b12] Londono M. A., Harmon C. L. & Polston J. E. Evaluation of recombinase polymerase amplification for detection of begomoviruses by plant diagnostic clinics. Virology journal 13, 48, doi: 10.1186/s12985-016-0504-8 (2016). 27000806 PMC4802622

[b13] Drummond T. G., Hill M. G. & Barton J. K. Electrochemical DNA sensors. Nature biotechnology 21, 1192–1199, doi: 10.1038/nbt873 (2003). 14520405

[b14] Lin C. S. . An electrochemical biosensor for the sensitive detection of specific DNA based on a dual-enzyme assisted amplification. Electrochim Acta 147, 785–790, doi: 10.1016/j.electacta.2014.10.092 (2014).

[b15] Yola M. L., Eren T. & Atar N. A novel and sensitive electrochemical DNA biosensor based on Fe@Au nanoparticles decorated graphene oxide. Electrochim Acta 125, 38–47, doi: 10.1016/j.electacta.2014.01.074 (2014).

[b16] Wang Z. . Electrocatalytic oxidation of phytohormone salicylic acid at copper nanoparticles-modified gold electrode and its detection in oilseed rape infected with fungal pathogen Sclerotinia sclerotiorum. Talanta 80, 1277–1281, doi: 10.1016/j.talanta.2009.09.023 (2010). 20006087

[b17] Vetrone S. A., Huarng M. C. & Alocilja E. C. Detection of Non-PCR Amplified S. enteritidis Genomic DNA from Food Matrices Using a Gold-Nanoparticle DNA Biosensor: A Proof-of-Concept Study. Sensors-Basel 12, 10487–10499, doi: 10.3390/s120810487 (2012). 23112611 PMC3472839

[b18] Kleijn S. E. F., Lai S. C. S., Koper M. T. M. & Unwin P. R. Electrochemistry of Nanoparticles. Angew Chem Int Edit 53, 3558–3586, doi: 10.1002/anie.201306828 (2014). 24574053

[b19] Riley D. J. Electrochemistry in nanoparticle science. Curr Opin Colloid In 7, 186–192, doi: Pii S1359-0294(02)00047-X, Doi 10.1016/S1359-0294(02)00047-X (2002).

[b20] Luo X. L., Morrin A., Killard A. J. & Smyth M. R. Application of nanoparticles in electrochemical sensors and biosensors. Electroanal 18, 319–326, doi: 10.1002/elan.200503415 (2006).

[b21] Lin L., Liu Y., Tang L. H. & Li J. H. Electrochemical DNA sensor by the assembly of graphene and DNA-conjugated gold nanoparticles with silver enhancement strategy. Analyst 136, 4732–4737, doi: 10.1039/c1an15610a (2011). 21952074

[b22] Liu G. D. . Aptamer-Nanoparticle Strip Biosensor for Sensitive Detection of Cancer Cells. Anal Chem 81, 10013–10018, doi: 10.1021/ac901889s (2009). 19904989 PMC2814445

[b23] Wang W. T., Fan X. J., Xu S. H., Davis J. J. & Luo X. L. Low fouling label-free DNA sensor based on polyethylene glycols decorated with gold nanoparticles for the detection of breast cancer biomarkers. Biosens Bioelectron 71, 51–56, doi: 10.1016/j.bios.2015.04.018 (2015). 25884734

[b24] Iacobellis N. S. Pseudomonas syringae and related pathogens: biology and genetic. (Kluwer Academic Publishers, 2003).

[b25] Katagiri F., Thilmony R. & He S. Y. The Arabidopsis thaliana-pseudomonas syringae interaction. The Arabidopsis book/American Society of Plant Biologists 1, e0039, doi: 10.1199/tab.0039 (2002). PMC3243347 22303207

[b26] Trusov Y. . Heterotrimeric G proteins facilitate Arabidopsis resistance to necrotrophic pathogens and are involved in jasmonate signaling. Plant Physiol 140, 210–220 (2006). 16339801 10.1104/pp.105.069625 PMC1326045

[b27] Wee E. J. H., Lau H. Y., Botella J. R. & Trau M. Re-purposing bridging flocculation for on-site, rapid, qualitative DNA detection in resource-poor settings. Chem Commun 51, 5828–5831, doi: 10.1039/c4cc10068a (2015). 25622026

[b28] Frens G. Controlled Nucleation for Regulation of Particle-Size in Monodisperse Gold Suspensions. Nature-Phys Sci 241, 20–22 (1973).

[b29] Hawkins T. L., Oconnormorin T., Roy A. & Santillan C. DNA Purification and Isolation Using a Solid-Phase. Nucleic acids research 22, 4543–4544, doi: 10.1093/nar/22.21.4543 (1994). 7971285 PMC308491

[b30] Ng B. Y. C. . Rapid, Single-Cell Electrochemical Detection of Mycobacterium tuberculosis Using Colloidal Gold Nanoparticles. Anal Chem 87, 10613–10618, doi: 10.1021/acs.analchem.5b03121 (2015). 26382883

