# Supplementary material for: Specific and Sensitive Isothermal Electrochemical Biosensor for Plant Pathogen DNA Detection with Colloidal Gold Nanoparticles as Probes
Source: Sci Rep. 2017 Jan 17;7:38896. doi: 10.1038/srep38896 (PMC5240331; doi:10.1038/srep38896)
Supplement: Supplementary Information [file srep38896-s1.pdf]

Specific and Sensitive isothermal Electrochemical Biosensor for Plant Pathogen DNA Detection with Colloidal Gold Nanoparticles as Probes

Han Yih Lau[1,2,§], Haoqi Wu[1,3,§], Eugene, J.H. Wee[1], Yuling Wang[1,]*, Jose R. Botella[2,]* and Matt Trau[1,4,]*

[1]Centre for Personalized Nanomedicine, Australian Institute for Bioengineering and Nanotechnology, The University of Queensland, Australia

[2]Plant Genetic Engineering Laboratory, School of Agriculture and Food Sciences, The University of Queensland, Australia

[3]Department of Macromolecular Science, National Key Laboratory of polymer engineering, Fudan University, Shanghai, 200433, China

[4]School of Chemistry and Molecular Biosciences, The University of Queensland, Brisbane QLD 4072, Australia

[§]Authors contributed equally.

Supplementary Information

Fig S1: TEM image of as-prepared gold nanoparticles (AuNPs) used in this study.

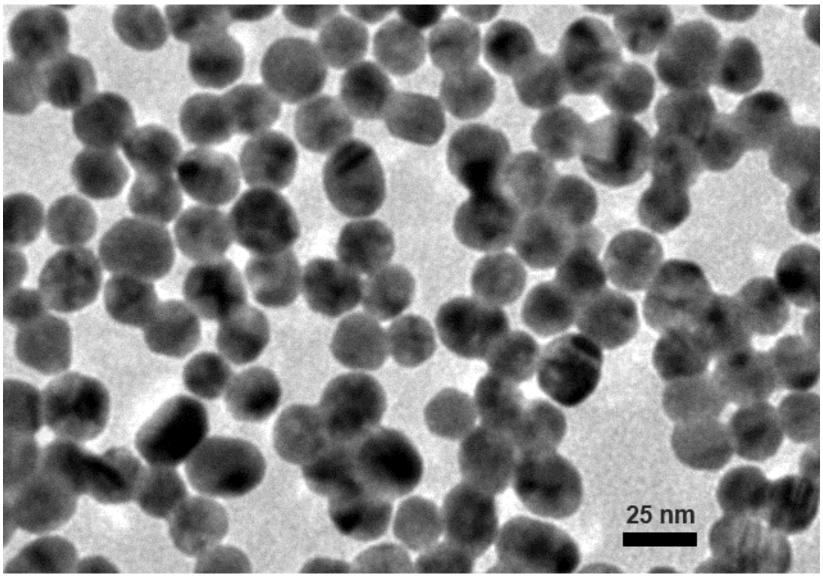

Fig S1: TEM image of as-prepared gold nanoparticles (AuNPs) used in this study.

Fig S2: Specificity study for plant pathogen DNA detection. (A) DPV curve and (B) Current-response to *P. syringae* (Psy), *Botrytis cinerea* (Bot) and *Fusarium oxysporum* f.sp. *conglutinans* (Foc) as well as a no template control (NTC). Error bars represent ±SD, n = 3. (C) Electrophoresis gel image of PCR products.

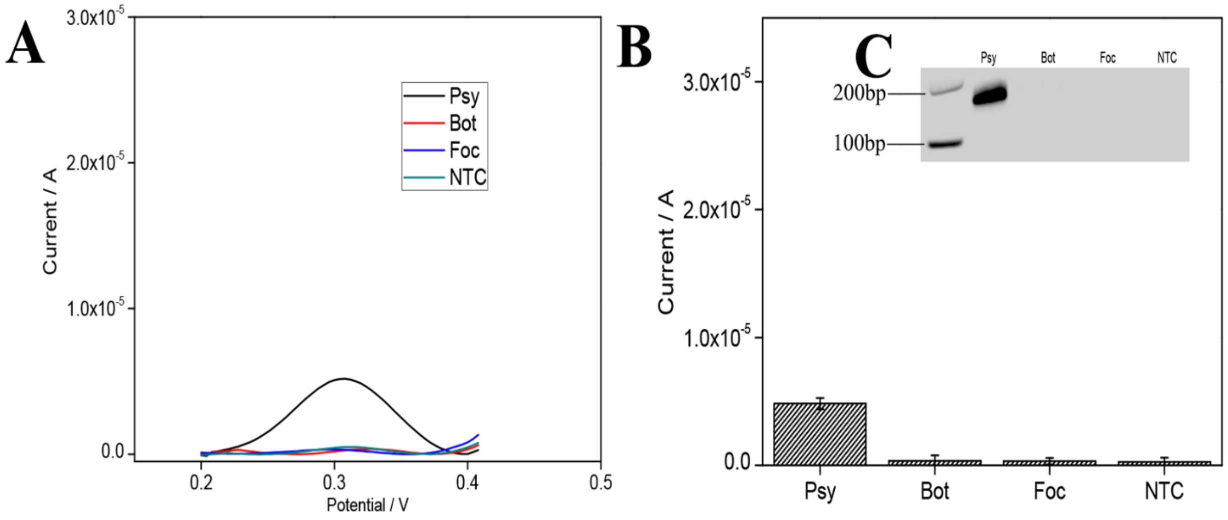

Fig S2: Specificity study for plant pathogen DNA detection via electrochemical assay after PCR amplification.

Fig S3: qPCR quantification of *P.syringae* (Psy) in 1 ng of extracted DNA from leaves at various stage 1 and stage 2 infections. The amount of pathogen DNA was estimated from calibration plot of known target concentrations.

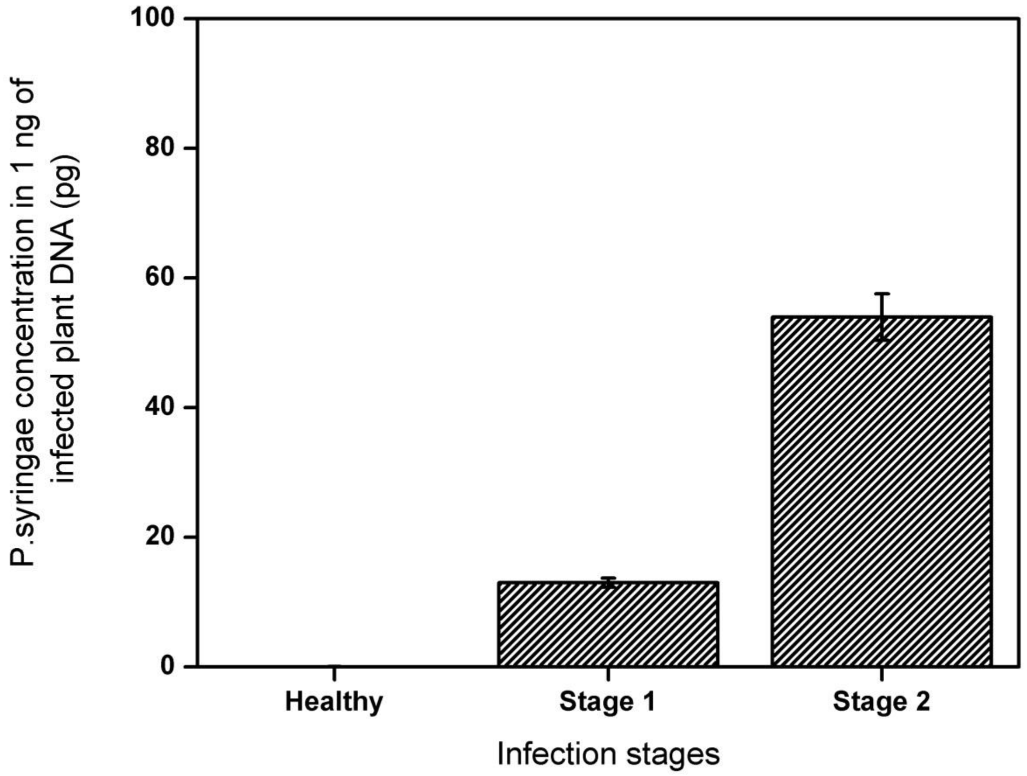

Fig S3: qPCR quantification of *P.syringae* (Psy) in 1 ng of extracted DNA from leaves at various stage 1 and stage 2 infections.
